# How COVID-19 and other pathological conditions and medical treatments activate our intravascular innate immune system

**DOI:** 10.3389/fimmu.2022.1030627

**Published:** 2023-02-03

**Authors:** Bo Nilsson, Oskar Eriksson, Karin Fromell, Barbro Persson, Kristina N. Ekdahl

**Affiliations:** ^1^ Department of Immunology, Genetics and Pathology (IGP), Uppsala University, Uppsala, Sweden; ^2^ Linnæus Center of Biomaterials Chemistry, Linnæus University, Kalmar, Sweden

**Keywords:** leukocytes, platelets, blood cascade systems, intravascular innate immune system, cross talk

## Abstract

COVID-19 has been shown to have a multifaceted impact on the immune system. In a recently published article in Front Immunol, we show that the intravascular innate immune system (IIIS) is strongly activated in severe COVID-19 with ARDS and appears to be one of the causes leading to severe COVID-19. In this article, we describe the IIIS and its physiological function, but also the strong pro-inflammatory effects that are observed in COVID-19 and in various other pathological conditions and treatments such as during ischemia reperfusion injury and in treatments where biomaterials come in direct contact with blood in, e.g., extracorporeal and intravasal treatments. In the present article, we describe how the IIIS, a complex network of plasma proteins and blood cells, constitute the acute innate immune response of the blood and discuss the effects that the IIIS induces in pathological disorders and treatments in modern medicine.

## 1 Introduction

The blood consists of a large number of plasma proteins that form our innate immune barrier with regard to the recognition and destruction of microorganisms. These plasma proteins are part of the intravascular innate immune system (IIIS). The IIIS as a composite entity was first discussed by Engelman and Massberg (1), but in the present article we widen the concept to include most of the innate immune components of the blood and discuss the effects that the IIIS induces in pathological disorders and treatments in modern medicine. The IIIS discussed in the present contribution, consists of the blood cascade system (the complement, the coagulation, the kallikrein/kinin [contact system], and the fibrinolytic systems) and individual proteins such as collectins, pentraxins, etc. In addition, blood cells such as granulocytes (PMNs), monocytes and platelets are essential effector cells in this crosstalk (2). Over the years, research in these areas has been very segregated. Although "cross-talk" between the different cascade systems has been demonstrated, many potential interactions have been overlooked due to technical reasons.

Human blood has often been used by researchers for studies of the IIIS in the various disciplines. Since the blood needs to be anticoagulated in order to be able to separate the plasma/serum from the blood cells, various techniques have been used. The complement and the coagulation cascades depend on divalent cations such as Ca^2+^ and Mg^2+^ ions, to function. Therefore, citrate that chelates Ca^2+^or EDTA that chelates both Ca^2+^ and Mg^2+^ ions, have been used. However, this has the consequence that not only is the coagulation system turned off, but also that the blood's ability to activate complement is hindered. Traditionally, therefore, functional complement studies have been done with serum (i.e., the remaining liquid phase after the blood has coagulated), while for the coagulation, kallikrein/kinin, and fibrinolysis systems, citrate plasma reconstituted with Ca^2+^ has been used. In serum, the entire coagulation system, and parts of the kallikrein/kinin and fibrinolysis systems are activated (and the intact proteins partially activated and consumed), which means that cross-talk studies with the complement system do not work. Citrate, on the other hand, leads to an impact on the function of the complement system even after Ca^2+^ restitution. If cells are included in this equation, it becomes even more complicated. This has led us and other colleagues to start to do studies in whole blood, often completely without anticoagulation. This can be achieved by coating the walls of test tubes or tubing with heparin or specially made polymers that prevent activation of the coagulation system (3). With these types of systems, it has been possible to demonstrate a number of “new” interactions between the various systems, which is important for understanding IIIS's function and regulation. It is time to seriously consider the components of the blood that constitute the acute innate immune response as a common system and not study the different parts separately, especially with regard to the immune functions.

## 2 IIIS structure

**Figure 1:**
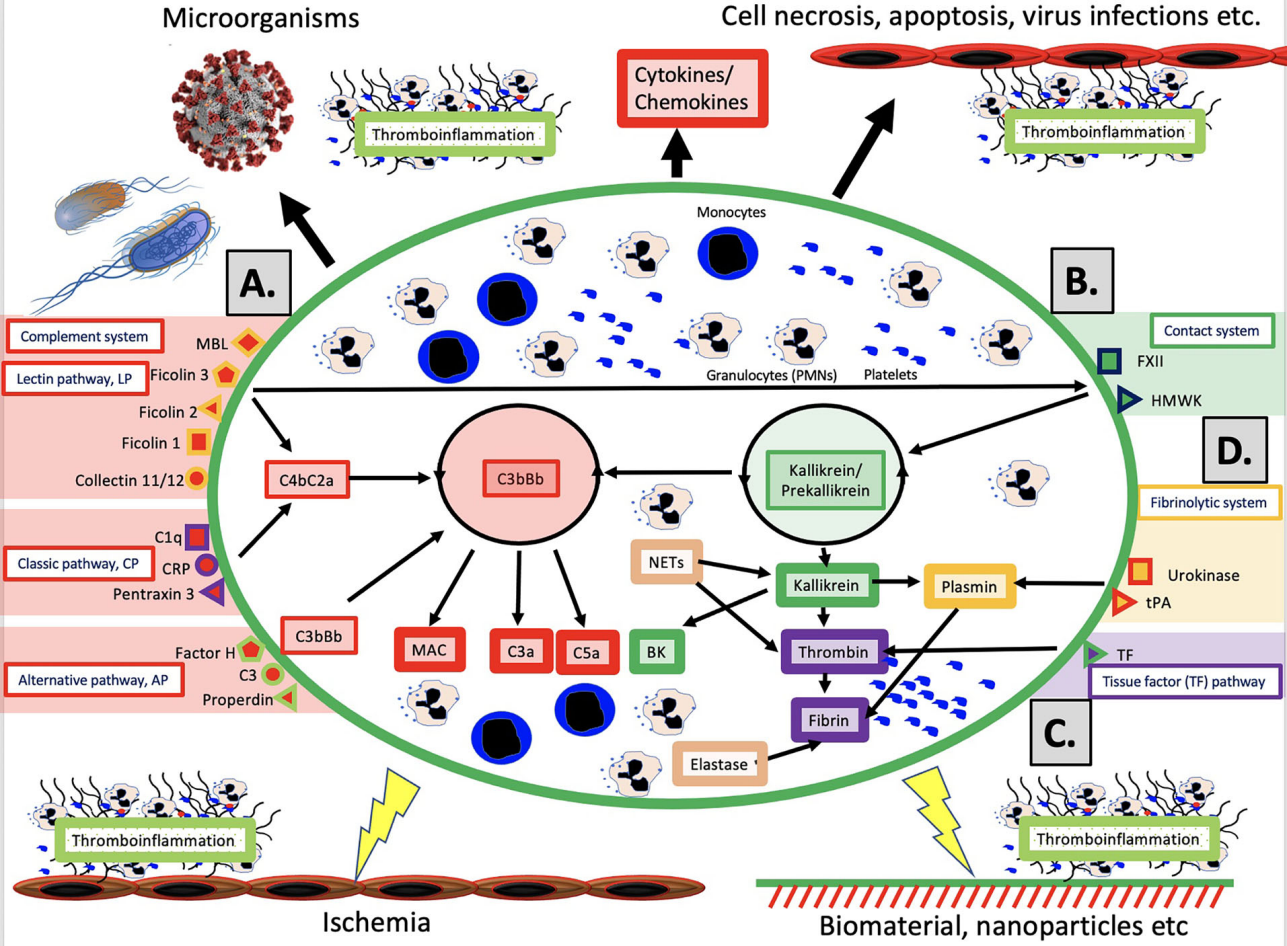
Overview of the intravascular innate immune system, IIIS. The oval encompasses the IIIS which comprises both blood cells (monocytes, granulocytes [PMNs], platelets) and plasma cascade systems. The four main cascades are the complement system **(A)**; the contact (or kallikrein/kinin) system **(B)**; the tissue factor (TF) or coagulation pathway **(C)**; and the fibrinolytic system **(D)**. **(A)**: Complement is activated by the lectin pathway (LP) *via* target recognition by mannose binding lectin (MBL), Ficolins 1, 2, 3 or Collectins 11/12, by the classical pathway (CP) *via* C1q, CRP or Pentraxin 3, or by the alternative pathway (AP) which may be initiated by direct binding of Factor H, C3 or properdin, but whose main function is as a potent amplifier. Recognition leads to assembly and activation of the LP/CP convertase (C4aC2b) and C3bBb (AP) both of which cleave C3 to the anaphylatoxin C3a and initiates formation of the membrane attack complex (MAC) and the anaphylatoxin C5a (more potent than C3a). **(B)**: The contact system is activated when its main recognition molecule Factor(F)XII autoactivates after binding to negatively charged moieties, e.g., glycosaminoglycans or matrix proteins. Together with high molecular weight kininogen (HMWK) and prekallikrein, an amplification loop is formed which leads to the potent proinflammatory and angiogenetic mediator bradykinin (BK). **(C)**: The main initiator of coagulation *in vivo* is tissue factor (TF) which under physiological conditions is absent in blood, but when exposed it triggers the external pathway of coagulation generating high amounts of thrombin, leading to platelet activation and fibrin formation. **(D)**: The fibrinolytic system is initiated when the zymogen plasminogen is turned into active plasmin by urokinase or tissue plasminogen activator (tPA). Plasmin dissolves solid fibrin clots into soluble peptides. Taken together activation of the IIIS generates thromboinflammatory reactions which may be triggered by physiological stimuli such as invading microorganisms or turn-over of autologous cells by necrosis or other mechanisms as depicted in the top of the figure. Similar thromboinflammation is also triggered by different medical treatment modalities such as ischemia during transplantation, or extracorporeal or intravascular treatment with various forms of biomaterials or pharmacological nanoparticles. These reactions may counteract the results of these treatments and cause harm to the patient as discussed in the text. Figure from (4). Published with open access under the Creative Commonas CC-BY licence.

The schematic structure of the IIIS is described in detail in **Figure 1**. The core of the IIIS consists of the cascade systems in the blood, i.e., the complement, coagulation, contact and fibrinolytic systems. The reason for mentioning them first is that they contain many of the recognition molecules that activate the entire IIIS. In the figure, they are marked as parts of the activation pathways initiated by the various cascade systems. The complement system has three defined activation pathways; the classical (CP), the alternative (AP) and the lectin pathway (LP) (5, 6). CP is initiated by C1q which binds to IgG and IgM in immune complexes, and also to bound CRP and pentraxin 3. LP is activated by several lectins (i.e., carbohydrate-binding proteins) such as MBL, Ficolin-1, -2, and -3, as well as by the collectins 11/12. AP functions primarily as an amplification loop but can be specifically activated by properdin together with C3 with factor H as the key “recognition” regulator.

*In vivo*, it has traditionally been considered that the coagulation system is activated by the so-called the extrinsic pathway *via* the protein Tissue Factor (TF) which is exposed in the vessel wall in case of endothelial damage and may be expressed in most cells outside the bloodstream during inflammation. TF can also be expressed by monocytes and on the endothelium during inflammatory reactions, and active TF exposed to blood leads to strong coagulation activation. The contact system is an alternative pathway for coagulation activation that is initiated upon contact between blood and foreign surfaces (7). The contact system is the reason why blood without anticoagulants clots in a test tube. The contact system is activated by Factor XII (FXII), and its activation and amplification loop constitute the start of the internal (intrinsic) activation pathway of the coagulation system. The contact system probably has a limited role for physiological hemostasis, which is illustrated by the fact that FXII deficiency does not lead to an increased tendency to bleed. However, it affects the amplitude of the clot , which has led to that inhibition of FXII is being investigated as a new strategy for antithrombotic drugs . the contact system also leads to increased inflammation *via* the production of mediators such as bradykinin (BK) (8). As part of the innate immune system, FXII is able to bind to microorganisms and apoptotic and damaged cells, thereby containing the target in fibrin, which can be phagocytized by innate immune cells (7).

The fibrinolytic system is started by urokinase, tissue plasminogen activator (tPA) and FXIIa by activating the zymogen plasminogen to plasmin (9). Activated plasmin in turn breaks down the fibrin networks formed in the final step of the coagulation cascade, thus acting as a physiological limiting mechanism that digests clotted blood after the healing process has begun and limits its spread. A significant cross-activation can also occur directly or indirectly *via* leukocytes and platelets, causing activation of a cascade system to spread to the entire IIIS (10, 11).

## 3 Thromboinflammation as part of IIIS function

The cascade systems of the blood are evolutionarily ancient, which can be explained by the fact that they not only, but above all, function as "garbage handling systems". Not only the recognition and elimination of microorganisms, but also transportation of foreign substances and particles form a large part of the function. Here, removal of apoptotic and necrotic cells is an important task. Furthermore, IIIS activation is the starting point for healing in connection with tissue damage where the debris handling function is an important part together with the release of growth factors from, e.g., activated platelets. The proteins in the various cascades have common original proteins and it is often possible to identify the mother molecules in evolutionarily early organisms. The systems have then developed through gene duplications either through the global gene duplications that occurred during evolution or through gene duplications occurring locally, which creates clusters of genes with similar function. As an example, complement factor C3 can illustrate the gene duplication phenomenon (12). A C3-like molecule is found early in evolution and after two genome duplications, C3, C4, C5 and also the proteinase inhibitor α_2_M (with a similar functional mechanism) are now found in vertebrates (13). Local duplications have led to the cluster of complement inhibitors found on chromosome 1 that encodes factor H, FHL-1, FHRs, DAF, MCP, C4BP, etc (14). Similar duplications have occurred within the other cascade systems, which is the reason why complicated cascade systems have been possible to evolve.

95% of all current animal species rely solely on the innate immune systems which in vertebrates largely consist of IIIS (15). Only 5% of currently living animal species have an adaptive immune system with T and B cells. The IIIS has a specific, preset function to distinguish self from non-self that is always available and does not need to be trained. Unlike the adaptive immune system, IIIS can only distinguish self from non-self at the species level and not at the individual level, meaning that it can react against its own autologous cells, which is relevant to its role in disease and treatment.

The physiological thromboinflammation that results from the reactions described above, stops bleeding by platelet aggregation and fibrin formation and cleans up the damaged tissue by activating leukocytes and platelets while coordinating healing of the tissue, etc. In a similar way, IIIS reacts to infections with different types of microorganisms. In these reactions, IIIS helps to kill the microorganisms or the infected cells, after which the tissue is cleared and healed as a result of IIIS functions. Sometimes, however, the reaction can overshoot the target and become far too strong, leading to severe inflammation in the tissue. The end result is then a thromboinflammation with activation of all IIIS components.

## 4 COVID-19

In the case of COVID-19, the entire IIIS is strongly activated, especially in those patients who are admitted to the intensive care unit with ARDS-like symptoms. In 2020, we studied 65 patients with intensive care-requiring COVID-19 and have then been able to observe that these patients have a pronounced activation of IIIS, with activation of all cascade systems, especially the kallikrein/kinin system, which is heavily consumed (activated) (4, 6). The cause of the activation is likely multifactorial. The virus itself can activate IIIS directly *via* CP and LP of the complement system and *via* the contact system (16). More likely, it is the tissue damage in the lungs with apoptotic and necrotic cells that initiates this overactivation of IIIS in its efforts to eliminate cells (6). The activation of IIIS has prognostic significance and strong activation of prekallikrein and high molecular weight kininogen (HMWK) on arrival to the ICU has been shown to have prognostic significance for gas exchange in the lungs and for mortality during the continued course of care (4). The mechanism which causes impaired oxygenation may be related to the fact that the IIIS activation leads to the formation of the inflammatory mediators C5a and BK which in turn causes leukocyte infiltration with neutrophil granulocytes and monocytes as well as increased vascular permeability, resulting in leakage of plasma proteins such as fibrinogen into the alveoli and an increased diffusion distance between pneumocytes and endothelial cells (6). The poor oxygenation can lead to further ischemia, which in turn causes even more IIIS activation thereby creating a vicious cycle.

## 5 IIIS activation in therapeutic medicine

**Figure 2:**
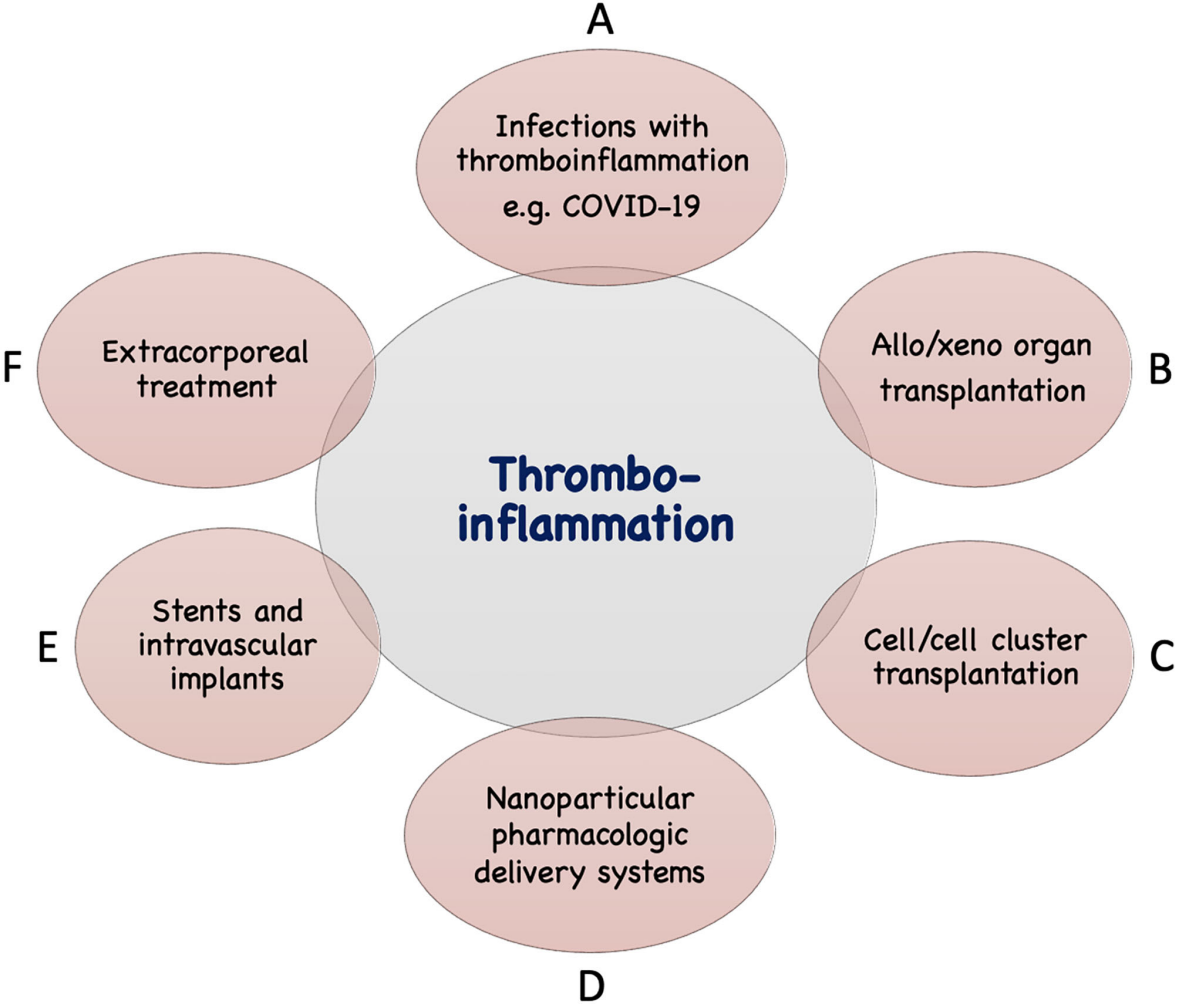
Examples of conditions and treatments which involve tromboinflammation. Activation of IIIS leading to thromboinflammatory reactions which may be detrimental occur in a number of clinical conditions, as well as treatments in general clinical practice. These include different infections such as with COVID-19 or sepsis **(A)**, transplantation: either of solid organs in allogeneic or xenogeneic setting **(B)** or of cells or cell clusters, e.g., islets of Langerhans **(C)**, drug distribution using nanoparticles in the body **(D)**, treatment with biomaterials, e.g., stents, intravascular devices **(E)**, and extracorporeal treatment such as, hemodialysis, cardiopulmonary treatment, ECMO, plasmapherisis etc **(F)**.

In addition to the IIIS activation described above seen in infections such as COVID-19, thrombotic conditions (cardiac infarction and stroke) and various treatment modalities used in the clinic today are associated with IIIS activation and thromboinflammatory reactions which may seriously interfere with the results of these treatments. The mechanisms involved are direct IIIS attack against cell surfaces, e.g., in ischemia reperfusion injury (IRI) and against artificial/solid materials in different treatment modalities. Such treatment procedures include various types of transplantations, employing nanoparticles and liposomes as drug delivery systems, and the use of different biomaterials in intravascular and extracorporeal applications as described in **Figure 2** and discussed in the following sections.

## 6 Extracorporeal circulation

The properties of IIIS mean that it reacts with anything that is not the species’ own tissue, also including artificial materials. This is a problem in extracorporeal treatments such as hemodialysis, cardiopulmonary bypass, extracorporeal mechanical oxygenation (ECMO), plasmapheresis, etc. The activation of IIIS on biomaterials occurs through binding/adsorption of plasma proteins to the material surface. This interaction leads to the formation of a single layer of proteins on the biomaterial surface. When a protein binds to a material surface (metal, polymer, plastic, etc.), the shape of the protein changes. Certain proteins are thereby artificially activated so that the cascade systems are in turn activated (17, 18). The typical example is FXII, which is spontaneously activated to FXIIa on glass and certain metals, e.g., titanium, thereby activating the contact system (19). But also complement factor C3 can activate the AP and surface-bound IgG the CP (18, 20). However, specific activation takes place in exceptional cases, e.g., so that the LP is activated by ficolin-2 when it binds to hemodialysis filters (21). The consequence of these reactions is that there always is a need to use anticoagulants in connection with this type of treatments to avoid clot formation in the circuits. Citrate is sufficient for plasmapheresis, but high- or low-molecular-weight heparin is required for hemodialysis, heart-lung machine treatment and ECMO (22). In the later treatments, you can also use heparin-coated filters and hose sets (23). Despite this, IIIS is activated by the treatment. In hemodialysis, the material surfaces of tubes and filters form activation surfaces for both the kallikrein/kinin and complement systems, which leads to that the material surfaces being attacked by platelets, granulocytes and monocytes and to the formation of the highly inflammatory peptides C5a and BK (17, 24). Interestingly, cascade system activation also occurs on gas surfaces in liquids, such as in heart-lung machines and ECMO devices if the gas surface is exposed (25, 26). In summary, a whole-body inflammation occurs which can spread to inflammation in the vessel walls of the treated patients. This may be a part of the reason for the greatly increased risk of arteriosclerosis and infections in dialysis patients, as well as the fact that the median survival in hemodialysis treatment is only 4 years (27). With heart-lung machine treatment and ECMO, thromboembolic complications are common and despite both soluble and surface-bound heparin, complement and kallikrein/kinin system activation occurs. This leads to platelet activation and TF expression on leukocytes, which contributes to further thrombus formation.

## 7 Ischemia/reperfusion injury

A condition that breaks the self/non-self barriers in disease and treatments is ischemia, i.e., lack of oxygen in cells and tissues. Ischemia is a major stress for the cell, which can lead to changes in cell membrane composition and protein expression. The condition can worsen through further cellular stress when oxygenation and blood flow are restored, leading to so-called ischemia/reperfusion injury (I/R injury; IRI) (6, 28).

Consequently, IIIS that come into contact with a cell exposed to hypoxia can recognize the cell surface as foreign, causing a thromboinflammatory reaction and, by extension, IRI and cell death. IRI damage occurs during reperfusion of a transplanted organ, or the tissue area exposed to ischemia as a result of a thromboembolism, e.g., during reperfusion as a result of percutaneous coronary intervention (PCI) in the event of imminent myocardial infarction, stroke, etc. The ischemic cell has a different phenotype than the native, leading to IIIS recognition molecules in the complement and kallikrein/kinin systems recognizing the cell as foreign. Primarily, MBL and MASP-2 of the LP and natural IgM antibodies have been suspected to be the cause of IIIS activation (28). This means that when the thrombus is removed in connection with PCI or thrombolysis, in addition to the ischemic injury, there is an injury linked to the reperfusion of the ischemic area and which is mediated by IIIS. Heparin treatment has limited effect here.

Similarly, damage occurs during graft reperfusion. When the organ (kidney, liver, heart, pancreas, etc.) is removed from the donor after the patient has been declared brain or heart dead, it is stored at +4°C under ischemic conditions, which means that the organ is firstly damaged by the ischemia, but also that the endothelium is damaged so that the protection against IIIS attack in the vessel tree is destroyed (28). This protection is in the glycocalyx (GC) that covers the endothelial cells and that is fragmented and lost in connection with ischemia. The GC protection is partly mechanical but also consists of GC-bound regulators of IIIS components, e.g., antithrombin (AT), C1INH, factor H, C4BP, etc. When the organ is reperfused in connection with transplantation, an IRI also occurs, potentially leading to “delayed graft function” after transplantation, which means that the organ does not function immediately after it is transplanted. This is a poor prognostic sign for the graft in the long term.

## 8 Xenotransplantation

IIIS also constitutes one of the fundamental obstacles in xenotransplantation. Xenotransplantation involves transplanting organs or cells from animals to humans. The aim is to be able to transplant porcine organs, e.g., kidneys, hearts, islets of Langerhans to humans. However, in connection with xenotransplantation, incompatibility between IIIS and the graft occurs on many levels due to that preformed antibodies activate the complement system and and regulators of IIIS do not function across species boundaries, consequently leading to xeno- graft rejection (29, 30). Recently, these barriers were crossed when a patient was transplanted with a bioengineered porcine graft (heart) with multiple knocked out xenoantigen genes and transgenically expressed complement regulators (31).

## 9 Cell transplantation

Cell therapy is an increasingly common treatment in healthcare. Cells infused directly into the bloodstream are subjected to a reverse ischemia/reperfusion injury that results in an activation of IIIS. These reactions occur to varying degrees upon infusion of isolated islets of Langerhans, liver cells or mesenchymal stromal cells (MSC) (32–34). The cells that are infused are not adapted to blood contact and expose more or less large amounts of extracellular matrix proteins on the surface, lack IIIS regulators that blood cells naturally have and are exposed to hypoxia, which induces ischemia and TF expression. Overall, this produces a thromboinflammatory reaction, which can damage the cell graft. Interestingly enough, this reaction also occurs during transplantation of own (autologous) tissue, which occurs during auto-transplantation of islets of Langerhans in association with pancreatitis, reflecting the specificity of the IIIS (35).

## 10 Treatments

In addition to the previously known treatments of IIIS activation *via* inhibition of the coagulation system (heparin, warfarin, direct oral anticoagulants [DOACs], etc), a large number of inhibitors have recently been developed to regulate the complement and contact systems. There are several licensed drugs that affect the components of the IIIS. Treatment of PNH (paroxysmal noctural hemoglobinuria), aHUS (atypical hemolytic uremic syndrome) and myasthenia gravis has for some time been possible by inhibition of complement factor C5 (anti-C5 antibody; eculizumab) with good results (5). Several other anti-complement drugs are under development for use in, for example, the treatment of COVID-19. One of these AMY-101 (C3 inhibitor of the compstatin family) which was recently shown to be able to provide faster clinical recovery and a lowering of several inflammatory parameters such as plasma levels of IL-6 (36). From the results of our recently published COVID-19 study, inhibition of the kallikrein/kinin system appears as a promising step in the search for therapeutic options for the treatment of COVID-19 ^15^. Drugs for the treatment of angioedema target the kallikrein/kinin system, where the effector BK can be inhibited by the BK receptor (BKR)-2 antagonist icantibant and the kallikrein-inhibiting antibody lanadelumab, both of which are registered orphan drugs in clinical use. The kallikrein inhibitor ecallantide, which is licensed in the USA, and both purified and recombinant C1INH are other possible examples of drugs that can potentially be used in IIIS activation in, e.g., COVID-19 patients (6). Successful treatment of ARDS with icatibant in hantavirus infection indicates that this may be the case and that the kallikrein/kinin system plays an important role in this type of condition and it is supported by the fact that a BKR2 inhibitor also alleviated another type of virus-induced ARDS (37).

## 11 Conclusion

The IIIS is a complex network of plasma proteins and blood cells that, in the present article, constitute most of the innate immune components of the blood. For technical reasons, the components of this network have been studied individually and the interactions between the individual components have not been fully revealed. By using whole blood models preferably without anticoagulants, the properties of the IIIS are possible to investigate *in vitro* and its reactions directed against ischemic cells in IRI and in intravascular and extracorporeal devices (biomaterials) possible to define.

## Data availability statement

The original contributions presented in the study are included in the article/supplementary material. Further inquiries can be directed to the corresponding author.

## Author contributions

BN has drafted, written and edited the manuscript. BP, OE, KF, KE have co-authored and edited the manuscript. All authors contributed to the article and approved the submitted version.
